# Thermal and Quantum Barrier Passage as Potential-Driven Markovian Dynamics

**DOI:** 10.1021/acs.jpcb.3c02744

**Published:** 2023-10-31

**Authors:** A. M. Zheltikov

**Affiliations:** Institute for Quantum Science and Engineering, Department of Physics and Astronomy, Texas A&M University, College Station, Texas 77843, United States

## Abstract

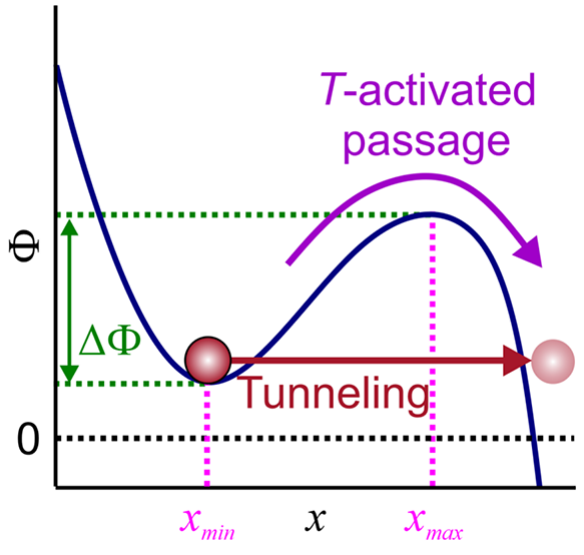

Rapidly progressing laser technologies provide powerful tools to study potential barrier-passage dynamics in physical, chemical, and biological systems with unprecedented temporal and spatial resolution and a remarkable chemical and structural specificity. The available theories of barrier passage, however, operate with equations, potentials, and parameters that are best suited for a specific area of research and a specific class of systems and processes. Making connections among these theories is often anything but easy. Here, we address this problem by presenting a unified framework for the description of a vast variety of classical and quantum barrier-passage phenomena, revealing an innate connection between various types of barrier-passage dynamics and providing closed-form equations showing how the signature exponentials in classical and quantum barrier-passage rates relate to and translate into each other. In this framework, the Arrhenius-law kinetics, the emergence of the Gibbs distribution, Hund’s molecular wave-packet well-to-well oscillatory dynamics, Keldysh photoionization, and Kramers’ escape over a potential barrier are all understood as manifestations of a potential-driven Markovian dynamics whereby a system evolves from a state of local stability. Key to the irreducibility of quantum tunneling to thermally activated barrier passage is the difference in the ways the diffusion-driving potentials emerge in these two tunneling settings, giving rise to stationary states with a distinctly different structure.

## 1 Introduction

Identifying the pathways whereby a system can overcome a binding potential, evolving from one state of local stability to another, is central to understanding a vast class of dynamic processes in physics, chemistry, and biology. Historic milestones en route toward attaining such understanding include the Arrhenius reaction-rate theory,^1^ Hund’s analysis of barrier penetration in molecular systems,^2,3^ Oppenheimer’s treatment of field-induced ionization,^4,5^ Fowler–Nordheim study of field-assisted electron emission from metals,^6,7^ Gamow–Gurney–Condon theory of alpha decay,^8−11^ Kramers’ work on thermally activated barrier crossing,^12^ and the Marcus theory of electron-transfer reactions in chemistry and biology,^13−15^ as well as the Keldysh theory of photoionization.^16^

Due to its extremely fast time scale and low activation energy, electron barrier passage serves as a universal trigger, starting a sequence of slower physical, chemical, or biological events.^17^ As one example, laser-driven electron tunneling has been shown to give rise to an ultrabroadband optical response,^18,19^ providing a source of high-order harmonics and attosecond field waveforms,^20^ as well as to enable a laser control of attosecond electron dynamics in solids.^21−27^ In its capacity as the fastest field-driven process, electron tunneling sets the physical limits for signal-processing speeds in laser–solid-interaction-based petahertz optoelectronics,^21−23,26^ the rates of chemical transformations, and efficiency of quantum control in physics, chemistry and biology.^28−33^

In living systems, over- and through-barrier electron transfer is central to reduction–oxidation reactions, driving ATP synthesis, cellular respiration, and intracellular energy flow.^34−37^ Proton tunneling, on the other hand, plays the key role in a vast variety of biological processes, ranging from transmembrane proton transfer to enzyme catalysis and intermediate metabolite isomerization.^38^ Understanding the barrier-passage dynamics behind such processes is thus central to the search for the physical underpinnings of life. The vision for such a search has been outlined by the greatest thinkers of the past,^39^ serving as a source of inspiration for generations of modern-age scientists. For many, including the author of this text, a deeper, in many ways eye-opening sense of this vision is a courtesy of Hiro-o Hamaguchi’s powerful and inspiring work on spectroscopic signatures of life.^40−42^

Rapidly progressing laser technologies provide powerful tools to study ultrafast charge-carrier barrier-passage dynamics in physical, chemical, and biological systems with an unprecedented resolution in space^43−48^ and time^20,23,33,49,50^ and a remarkable chemical and structural specificity.^51−56^ However, spanning across a remarkably broad range of fields and disciplines, from quantum physics,^57−59^ chemistry,^13,60^ and biology^34−37^ to stochastic models in genetics,^61,62^ the available theories of barrier passage operate with equations, parameters, and variables that are best suited for their respective areas of science. Making connections between these frameworks is often anything but easy. The treatment presented here aims to address this problem. We show that a model of a stochastic Markovian process as described by the Fokker–Planck equation (FPE) provides a unified framework for the description of a vast class of classical and quantum barrier-passage phenomena. That the FPE can be uniformly derived for both classical and quantum systems via a perturbative spectral analysis of pertinent Hamiltonians has been demonstrated by Bogoliubov and Krylov.^62^ The central idea of this study is to examine the FPE solutions applicable to classical and quantum systems in their capacity as descriptors of classical and quantum barrier-passage dynamics. We show that, in this framework, the Arrhenius-law kinetics, the emergence of the Gibbs distribution, Hund’s molecular wave-packet well-to-well oscillatory dynamics, Keldysh photoionization, and Kramers’ escape over a potential barrier are all understood as manifestations of a potential-driven Markovian dynamics whereby a system evolves from a state of local stability.

## 2 Barrier-Passage Potential Settings

**Figure 1:**
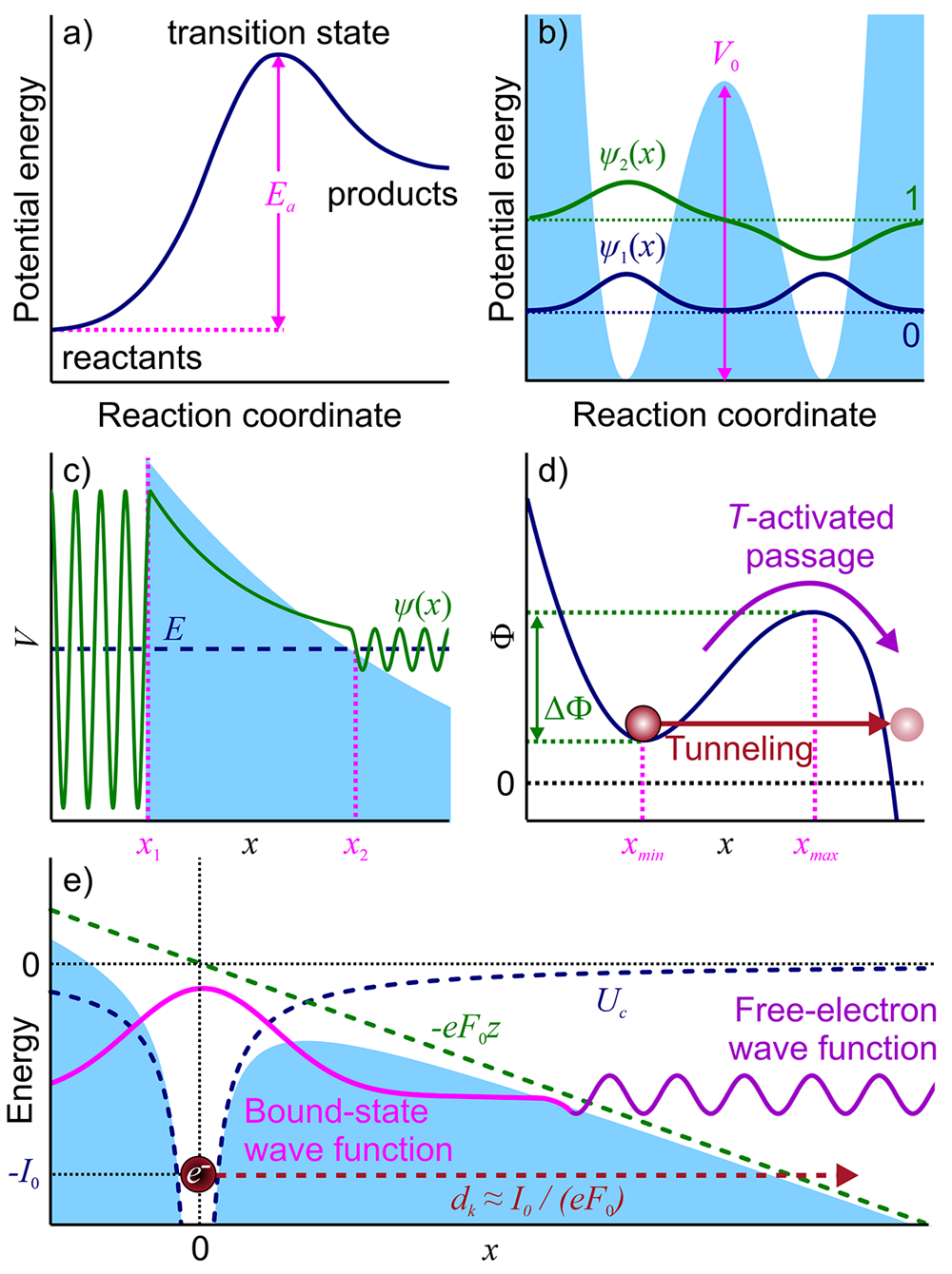
Potential energy profiles in signature settings of barrier-passage dynamics: (a) Arrhenius law kinetics in which a reactant (left of the barrier) overcomes an activation-energy barrier *E*_a_, giving rise to a buildup of a product state (right of the barrier); (b) reflection-symmetric double-well potential (blue shading) with a barrier of height *V*_0_ that separates two identical potential wells, giving rise to nearly degenerate doublets of energy eigenstates with symmetric and antisymmetric wave functions ψ_1_ (blue line) and ψ_2_ (green line) in Hund’s theory of molecular spectra; (c) potential energy *V*(*x*) (blue shading) and wave function (green line) in the Fowler–Nordheim–Gurney–Condon–Gamow tunneling of a quantum particle with energy *E* (blue dashed line), such that *E* < *V*(*x*) for *x*_1_ < *x* < *x*_2_, where *x*_*j*_ are the classical turning points, *V*(*x*_*j*_) = *E*, *j* = 1, 2; (d) potential energy profile allowing a Kramer’s escape over a potential barrier of height ΔΦ centered at *x*_max_ from a potential well centered at *x*_min_, giving rise to a thermally activated barrier passage (purple arrow) along with quantum tunneling across the barrier (red straight arrow); (e) laser-driven electron tunneling through a potential barrier of width *d*_K_ = *I*_0_/(*eF*_0_), formed by the binding potential of the atomic core *U*_c_ (blue dashed line) with an ionization energy *I*_0_ and an ac laser field with an amplitude *F*_0_, giving rise to the interaction energy of −*eF*_0_z (green dashed line).

As a starting point for this study, Figure 1 sketches generic potential profiles that give rise to signature barrier-passage dynamics in physical, chemical, and biological systems. Falling into this category are the potential-profile settings that enable the Arrhenius-law kinetics (Figure 1a), molecular wave packet well-to-well oscillations in Hund’s theory of molecular spectra (Figure 1b), electron emission and alpha decay in the Fowler–Nordheim, Gurney–Condon, and Gamow theories (Figure 1c), Kramer’s escape from a potential well (Figure 1d), and field-induced ionization in the Keldysh theory (Figure 1e). Below in this section, we provide a brief review of the key equations for the barrier-passage rates and times in these potential settings.

### 2.1 Arrhenius Law

In a generic setting typical of Arrhenius-law kinetics,^1^ a chemical reaction is viewed as a process in which reactants overcome an activation-energy barrier *E*_a_ (Figure 1a), which separates the reactant and product states. Thermally activated barrier passage in this setting gives rise to a buildup of a product state with a rate constant
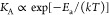
1where *T* is the temperature and *k* is the Boltzmann constant.

### 2.2 Hund’s Potential Setting

In Hund’s theory,^2,3^ the properties of molecules and their spectra are described in terms of double-well reflection-symmetric potentials binding atoms in molecules (Figure 1b). A barrier of finite height *V*_0_ separating two identical potential wells (Figure 1b), allows tunneling, which lifts the degeneracy of energy eigenstates with symmetric (ψ_1_ in Figure 1b) and antisymmetric (ψ_2_ in Figure 1b) wave functions. The superposition of ψ_1_ and ψ_2_ is a nonstationary state that shuttles back and forth from one well to the other with a beat period
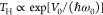
2where ω_0_ is the oscillation frequency in either of the potential wells.

### 2.3 Fowler–Nordheim–Gurney–Condon–Gamow Tunneling

In the Fowler–Nordheim model of field-induced electron emission, as well as in Gamow’s picture of alpha decay,^8^ developed, independently, by Gurney and Condon,^9,10^ a quantum-mechanical particle of mass *m* and energy *E* (an electron in the Fowler–Nordheim model or an alpha particle in the Gurney–Condon–Gamow theory) can penetrate a potential barrier (Figure 1c) with a transmission coefficient given by a signature exponential
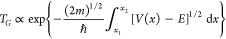
3Here, *V*(*x*) is the potential energy and *x*_*j*_ are the classical turning points where *V*(*x*_*j*_) = *E*, *j* = 1, 2.

### 2.4 Kramer’s Escape

Unlike quantum models by Hund, Fowler, Nordheim, Gurney, Condon, and Gamow, Kramers’ reaction rate theory^12^ treats an overbarrier escape of a system from a potential well (Figure 1d) as a thermally driven, noise-assisted process involving Brownian-motion-type dynamics. Starting with a diffusion equation for the dynamics of a reactive particle, this theory derives the following celebrated equation for the rate of thermally activated barrier crossing, broadly termed the Kramers escape rate:
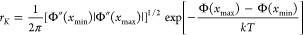
4Here, Φ(*x*) is the potential energy, *x*_min_ is the coordinate of the bottom of the potential well, and *x*_max_ is the coordinate of the top of the potential barrier (Figure 1d).

### 2.5 Keldysh Photoionization

The Keldysh theory of photoionization^16^ deals with laser-driven transitions between bound and quasi-free, Volkov-type electron states in the presence of a binding atomic potential (Figure 1e). As one of its central findings, this theory demonstrates that multiphoton ionization and electron tunneling are two pathways that dominate photoionization, respectively, in the weak- and strong-field regimes. The borderline between these regimes is defined in terms of the Keldysh adiabaticity parameter
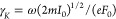
5where ω and *F*_0_ are the frequency and the amplitude of the driver field, *I*_0_ is the ionization potential, and *e* and *m* are the electron charge and mass. With γ_K_ > 1, multiphoton ionization plays a dominant role, while tunneling prevails when γ_K_ < 1. Over almost six decades, this insight from the Keldysh theory of photoionization has been pivotal to the research in strong-field laser science,^59,63^ providing a universal framework for a quantitative analysis of ionization in a remarkable diversity of light–matter interaction phenomena.^64−70^

### 2.6 In Search of a Unification

As can be seen from a brief review provided in the previous section, the available theories of barrier passage operate with equations, parameters, and variables that are best suited for a specific area of research or even a specific potential profile. Connections between these theories are far from obvious. Indeed, while the derivation of the Kramers escape rate and the Arrhenius law is based on the solution of classical stochastic equations, the Hund theory of molecular spectra, the Fowler–Nordheim–Gurney–Condon–Gamow (FNGCG) tunneling rate, and the Keldysh photoionization rate are all the products of quantum treatment. It is therefore far from clear whether the potential Φ(*x*) in Kramers’ escape rate and the reaction-activation energy *E*_a_ in the Arrhenius law have any relation to the potential *V*(*x*) in theories of quantum tunneling.

We show below in this paper that the relation between Φ(*x*) and *V*(*x*) is indeed nontrivial, as it is expressed by a Riccati differential equation and can be understood in terms of the pertinent Hamilton–Jacobi equation. With this relation in place, the exponential in Kramers’ escape rate [eq 4] will be shown to translate into the exponentials in the Arrhenius law [eq 1], Hund’s beat cycle [eq 2], and the FNGCG tunneling exponential [eq 3]. Providing a framework for deriving and understanding these relations is a unified treatment of classical and quantum barrier-passage phenomena that will be presented in this paper. This framework will be shown to reveal an innate connection between various barrier-passage phenomena and to provide useful insights into how the signature exponentials in the barrier-passage rates in eqs 1–5 relate and translate into each other.

## 3 The Fokker–Planck Equation

Central to our approach is a treatment of barrier-passage dynamics as a stochastic Markovian process, i.e., a stochastic process whose distribution function *w*(*x*, *t*) at a current moment of time *t* is fully determined by the initial distribution, *w*_0_(*x*) = *w*(*x*, *t* = *t*_0_), and can be found by solving a canonical Fokker–Planck equation (FPE)
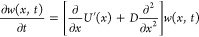
6with a diffusion coefficient *D* and potential *U*(*x*) serving as a driver for the diffusion process.

In a more general case of non-Markovian dynamics, the distribution function *w*(*x*, *t*) would depend on the entire prehistory, i.e., distributions at all of the earlier times *t*. The memory inherent in non-Markovian processes can be included by modifying eq 6 to the form of an integro-differential equation with time-dependent coefficients in front of both terms on its right-hand side and with integration over all the earlier times *t*.^71−74^

## 4 The Imaginary-Time Schrödinger Equation

In classical statistical mechanics, eq 6 is solved via a factorization^74^
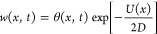
7where θ(*x*, *t*) is a solution to a generalized diffusion equation
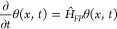
8with a Hamiltonian  and potential
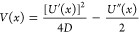
9With a variable-separation ansatz θ(*x*, *t*) = ψ(*x*) exp(−*λt*), eq 8 leads to an eigenfunction and eigenvalue problem
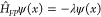
10Because *Ĥ*_*FP*_ is Hermitian, its eigenvalues *λ*_*n*_ are real. With a proper normalization, the eigenfunctions *ψ*_*n*_(*x*) of this operator satisfy the orthonormality relation ∫*ψ*_*n*_(*x*) *ψ*_*m*_(*x*) d*x* = *δ*_*nm*_, where *δ*_*nm*_ is the Kronecker delta.

eq 10 is the eigenvalue problem of the Schrödinger equation for a quantum system with a negative single-particle Hamiltonian evolving in imaginary time. Replacing time *t* in eq 8 by *t*′ = −*iℏt* and setting *D* = *D*_q_ = *ℏ*/2*m*, we arrive at the Schrödinger equation for a wave function ψ(*x*, *t*) in real time
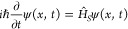
11where *Ĥ*_*S*_ = −*Ĥ*_*FP*_ is the single-particle Hamiltonian and the prime in time *t*′ is omitted.

## 5 Emergence of the Gibbs Distribution as a Large-*t* Limit

The Fokker–Planck equation [eq 6] can now be viewed as the continuity equation for the distribution *w*(*x*, *t*)
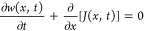
12with the probability current *J*(*x*, *t*) = *v*(*x*, *t*) *w*(*x*, *t*), current velocity *v*(*x*, *t*) = *b*(*x*) – *u*(*x*, t), drift *b*(*x*) = −*U*′(x), and osmotic velocity *u*(*x, t*) = *D*[ln *w*(*x, t*)]′^75−78^ It is straightforward to see that the stationary state of *w*(*x*, *t*), such that *∂w*/*∂t* = 0, is achieved when *v* = 0, i.e., *u*(*x*) = *b*(*x*) = −*U*′(*x*). Solving this equation for *w*(*x*, *t*) yields
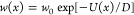
13With the potential *U*(*x*) and diffusion coefficient *D* understood in a sense of statistical mechanics, as *U*(*x*) = Φ(*x*)/(*mγ*) and *D* = *D*_t_ = *kT*/(*mγ*), where Φ(*x*) is a potential, *T* is the temperature, *m* is the mass, *k* is the Boltzmann constant, γ is the relaxation rate, eq 13 recovers the thermodynamic Gibbs distribution.

To see how this distribution emerges as a large-*t* limit of *w*(*x*, *t*), we first observe that, since ∫*ψ*_*n*_(*x*) *Ĥ*_*FP*_*ψ*_*n*_(*x*) d*x* = −*λ*_*n*_ = −∫[*∂ϕ*_*n*_(*x*)/*∂x*]^2^*D* exp[−*U*(*x*)] d*x* ≤ 0, with *φ*_*n*_(*x*) = *ψ*_*n*_(*x*) exp[*U*(*x*)/(2*D*)], the spectrum of eigenvalues *λ*_*n*_ is bounded from below by zero, *λ*_*n*_ ≥ 0. From *Ĥ*_*FP*_ exp[−*U*(*x*)/(2*D*)] = 0, we also find that ψ_0_(*x*) = *N*^1/2^ exp[−*U*(*x*)/(2*D*)], with *N* as dictated by normalization, is the ground-state eigenfunction that reaches the lower-bound eigenvalue λ_0_ = 0, thus translating into a stationary distribution *w*_0_ (*x*) = *N*^1/2^ exp[−*U*(*x*)/*D*)]. Whether a λ_0_ = 0 eigenfunction and, hence, a stationary distribution *w*(*x*) = *w*_0_(*x*), exist depends on the properties of the potential *U*(*x*) and the boundary conditions on *w*(*x*). Specifically, for natural boundary conditions, *J*(*x*, *t*) → 0 as |*x*| → ∞, the λ_0_ = 0 eigenfunction and the respective stationary distribution exist only when *U*(*x*) is positive and increases, at least asymptotically, as |*x*| → ∞.^71,74^

We can now represent the general solution to eq 8 as
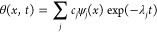
14with expansion coefficients defined from the initial condition θ_0_(*x*) = θ(*x*, *t* = *t*_0_) as *c*_*j*_ = ∫θ_0_(*x*) *ψ*_*j*_(*x*) d*x*. As long as the potential *U*(*x*) supports a ground state with λ_0_ = 0 and the first nonvanishing eigenvalue, λ_1_, is separated from λ_0_ by a nonzero gap Δλ = λ_1_ – λ_0_ ≠0, the distribution *w*(*x*, *t*), as can be seen from eqs 8 and 14, converges to
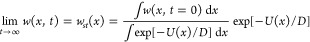
15as its large-*t* limit.^71,74^

It is readily seen from eqs 8, 14, and 15 that the lowest nonvanishing eigenvalue λ_1_ defines the large-*t w*(*x*, *t*) → *w*_st_(*x*) convergence rate. The inner product⟨θ(*x*, *t*)|θ(*x*, *t* = 0)⟩ = ∫*w*(*x*, *t*) d*x*, on the other hand, remains constant at any *t* as a generic expression of conservation of the number of particles.

## 6 Imposed and Emerging Potentials

With the solution to the Schrödinger equation [eq 11] written in the form of a generic Madelung decomposition, ψ(*x*, *t*) = [ρ(*x*, *t*)]^1/2^ exp[*i ms*(*x*, *t*))/ℏ], where ϕ(*x*, *t*) = *s*(*x*, *t*)/(2*D*_q_) = *ms*(*x*, *t*)/ℏ is the phase of ψ(*x*, *t*), and with the quantum probability density defined, in accordance with the Born rule,^57,79^ as ρ(*x*, *t*) = |ψ(*x*, *t*)|^2^, the continuity equation for ρ(*x*, *t*) reads
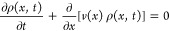
16where *v*(*x*, *t*) = *s*′(*x*, *t*) is readily interpreted as the local velocity of the quantum probability density current in the position space and is broadly referred to as the current, or advective velocity.^75−79^

With the drift *b*(*x*, *t*) defined now as the sum *b*(*x*) = *u*(*x*) + *v*(*x*) of the current velocity *v*(*x*, *t*) and the quantum analog of the osmotic velocity,^75−78^*u*(*x*, *t*) = *D*_q_(ln ρ)′, the continuity eq 16 can be rewritten as the Fokker–Planck equation for ρ(*x*, *t*):
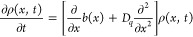
17We can now see that, similar to the probability distribution *w*(*x*, *t*) of a Markovian diffusion process, the quantum probability density ρ(*x*, *t*) satisfies the Fokker–Planck equation [cf. eqs 6 and 17]. Moreover, in both quantum physics and classical statistical mechanics, the Fokker–Planck equation emerges as an expression of the continuity of the respective probability distribution, *w*(*x*, *t*) and ρ(*x*, *t*). However, the way that the potential *U*(*x*) that drives the diffusion process behind the FPE emerges in classical and quantum physics is radically different. In classical statistical mechanics, this potential is given, or imposed, along with pertinent initial conditions, as a defining element of the overall physical, chemical, or biological setting. The potential *V*(*x*), on the other hand, enters into a thermally activated diffusion as a fictitious potential, as it is derivable from *U*(*x*) via eq 9.

By contrast, in quantum mechanics, it is the potential *V*(*x*) that is given, or imposed, as a part of the overall setting, defining, along with the initial conditions for ψ(*x*, *t*), the quantum evolution of a physical, chemical, or biological system. In such a setting, *U*(*x*) is emergent as a potential function whose negative gradient is the FPE drift, **b**(*x*) = −∇*U*(*x*), or, in a one-dimensional case, *b*(*x*) = −*U*′(*x*). With ψ(*x*) found from the Schrödinger equation [eq 11], the potential *U*(*x*) is fully defined, alongside the FPE drift *b*(*x*), by *U*′(*x*) = −*b*(*x*) = −(ℏ/*m*)*Re*[ψ′(*x*)/ψ(*x*)] – (ℏ/*m*)*Im*[(ψ′(*x*)/ψ(*x*)]. Thus, while *U*(*x*) is useful as it helps describe and understand the potential nature of the quantum probability density current, the FPE propagating the quantum probability density can be derived without an explicit knowledge of *U*(*x*).

To gain deeper insights into the emergence of the diffusion-driving potential *U*(*x*) in a quantum setting, we observe that, in addition to a diffusion-type equation for ρ(*x*, *t*) [eq 17], the Schrödinger equation [eq 11], in its capacity as an equation for a complex function ψ(*x*, *t*), leads to a Hamilton–Jacobi-type evolution equation^36−38^ for the phase *s*(*x*, *t*):
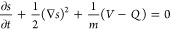
18Here, the potential function *Q* = ℏ^2^(2*m*)^−1^Δρ^1/2^/ρ^1/2^ = *m u*^2^/2 + (ℏ/2)∇·**u** is the de Broglie–Bohm quantum potential.^39,40^

**Figure 2:**
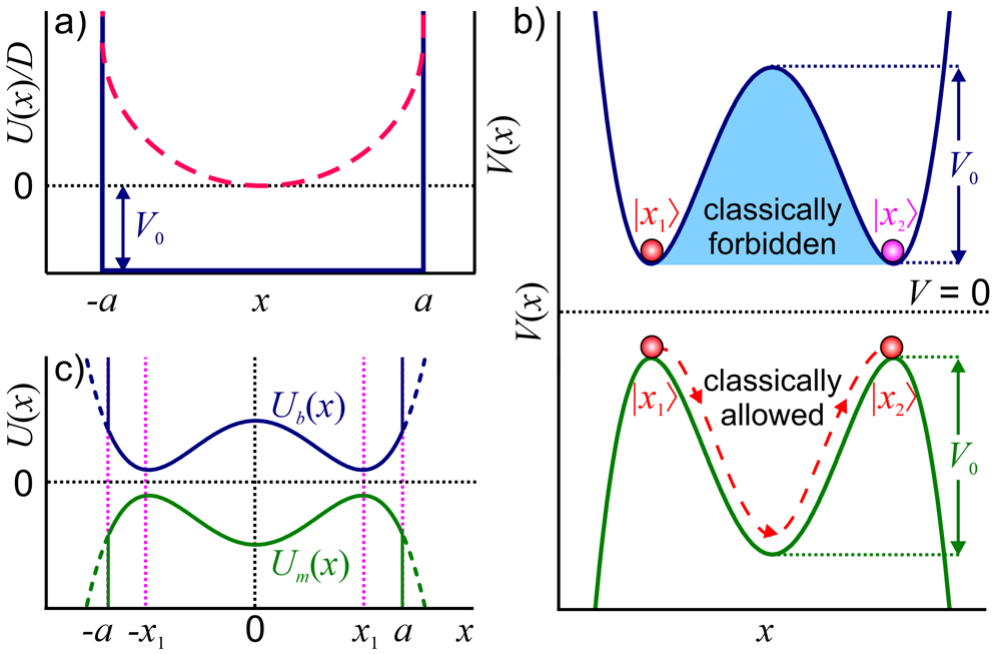
(a) Potential profile *V*(*x*) (blue solid line) for a quantum particle in an infinitely deep rectangular well, with *V*_0_ = *V*(0) = −π^2^*D*/(4*a*), along with the diffusion-driving potential *U*(*x*) (red dashed line), emerging for such a system via eq 19. (b) Bistable potential *V*(*x*) (blue line) of the barrier-passage problem with Minkowski action *S*_M_(*x*) and its inverted counterpart (green line), serving as a potential for Euclidean action *S*_E_(*x*) of the same problem. A search for the minimum of the Minkowski action for the initial and final states |*x*_1_⟩ and |*x*_2_⟩ (red circles) separated by a potential barrier yields no stationary path as the region under the barrier (shading) is classically forbidden. A search for the minimum of the Euclidean action leads to equations of motion for a classical particle in the inverted potential (green line), in which |*x*_1_⟩ and |*x*_2_⟩ (red circles) are connected by a classical path (red dashed line). (c) Bistable potential *U*_b_(*x*) (blue line) and its inverted, metastable counterpart *U*_m_(*x*) = −*U*_b_(*x*) (green line): general-form bistable and metastable potentials (dashed line) against bistable and metastable potentials with respectively reflecting and absorbing boundary conditions set at finite *x* = ±*a* (solid lines).

For an eigenstate of *Ĥ*_*S*_ with an energy eigenvalue *E* and *v* = 0, we find ∂*s*/∂*t* = −*E*/*m*. Equation 18 then leads to a Riccati-type differential equation relating the potential *V*(*x*) in the Schrödinger Hamiltonian *Ĥ*_*S*_ to the diffusion driver *U*(*x*):
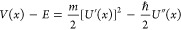
19In Figure 2a, we illustrate a potential profile *V*(*x*) for a quantum particle in an infinitely deep rectangular well along with the diffusion-driving potential *U*(*x*), emerging for such a system via eq 19.

## 7 Stationary States: Lifting the Gibbs-Distribution Curse

We are now in a position to observe that the means whereby the probability distributions ρ(*x*, *t*) and *w*(*x*, *t*) are stabilized in the FPE against, respectively, quantum evolution and thermally activated Brownian-motion-type dynamics are radically different. In the FPE pertaining to Brownian-motion dynamics [eqs 6 and 12], the current velocity *v*(*x*) is defined as the deviation of *u* = *D*(ln *w*)′ from −*U*′(*x*). The FPE solution *w*(*x*, *t*) is therefore stationary, *v*(*x*) = 0, and *∂w*/*∂t* = 0, only when *D*(ln *w*)′ = −*U*′(*x*), i.e., when eq 13 is satisfied.

In the FPE describing quantum evolution, on the other hand, a state is stationary, as one of the fundamental principles of quantum theory since Bohr’s 1913 breakthrough,^80−82^ whenever its wave function is an eigenfunction of the Hamiltonian *Ĥ*_*S*_. For such states, **v** = ∇*s* and ρ(*x*, *t*) = |ψ(*x*, *t*)|^2^ lead to *v*(*x*) = 0 and *∂ρ*/*∂t* = 0, lifting the curse of the Gibbs distribution as the large-*t* limit [eq 15]. Stationary states of this nature are in no way unique to quantum mechanics, but are shared by a vast variety of classical wave phenomena in electrodynamics, plasma physics, fluid dynamics, and oceanography,^83−89^ whose evolution is governed by the real-time Schrödinger equation [eq 11]. When they unfold in a suitable potential setting, e.g., as a quantum wave function tunneling through a potential barrier or as a localized mode of an electromagnetic field tunneling through a finite cladding of a waveguide, these wave processes, as they obey the same evolution equation, share the same archetype of tunneling dynamics.^90−92^ This dynamics, however, is distinctly different from the dynamics of thermally activated barrier passage (Figure 1d), as expressed by the FPE solution *w*(*x*, *t*) = θ(*x*, *t*) exp[−*U*(*x*)/(2*D*)], with θ(*x*, *t*) defined as a solution to the imaginary-time rather than the real-time Schrödinger equation [cf. eqs 8 and 11].

## 8 Bistable Potential, the Boltzmann Factor, and the Arrhenius Law

As a meaningful model of a potential that supports a ground state with λ_0_ = 0 and that helps understand the key aspects of barrier-passage dynamics in a vast class of physical, chemical, and biological systems, we consider a bistable double-well potential
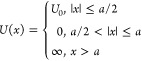
20In accordance with general properties of *Ĥ*_*FP*_ and *Ĥ*_*S*_ discussed in sections 4 and 5, the ground-state eigenvalue for such a potential, found by solving eq 10, is zero, λ_0_ = 0. The ground-state eigenfunction is^71,93^
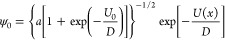
21The lowest nonvanishing eigenvalue is^71,74,93^
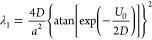
22For high *U*_0_ and low *D*, *U*_0_/(2*D*) ≫ 1, eq 22 gives
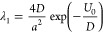
23With the potential *U*(*x*) and diffusion coefficient *D* understood in a sense of statistical mechanics, that is, as *U*(*x*) = Φ(*x*)/(*mγ*) and *D* = *kT*/(*mγ*), eq 23 recovers the Boltzmann factor
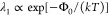
24with Φ_0_ = *mγU*_0_.

As shown in section 5, the lowest nonvanishing eigenvalue of *Ĥ*_*FP*_ defines the large-*t w*(*x*, *t*) → *w*_st_(*x*) convergence rate. We can now see from eq 24 that such a large-*t* rate of convergence to the Gibbs distribution in a bistable potential (eq 20) is λ_1_ ∝ exp[−Φ_0_/(*kT*)]. With the activation energy *E*_a_ in eq 1 identified with Φ_0_, as its natural assignment, eqs 23 and 24 recover the Arrhenius-law in eq 1.

## 9 Imaginary Time, Euclidean Action, and the Inverted Potential

To appreciate common statistical-mechanic underpinnings of classical and quantum barrier-passage phenomena, we consider the partition function
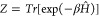
25where *Ĥ* is the Hamiltonian and β = 1/(*kT*).

With *Ĥ* = *p̂*^2^/(2*m*) + *V*(*x*), eq 25 gives
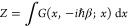
26where
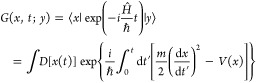
27is the transition amplitude and *D*[*x*(*t*)] stands for path integration.^94−96^ The transition amplitude *G*(*x*, *t*; *y*), also referred to as a propagator, defines the probability that a quantum system that starts from state |*y*⟩ at *t* = 0 will be found in state |*x*⟩ at the moment of time *t*.

With a new time variable introduced via Wick’s rotation, τ = *it*/*ℏ*, paralleling transformation from the real-time Schrödinger equation to its imaginary-time counterpart [see section 4], eq 27 yields
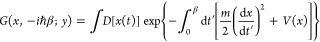
28Equation 26 is now seen to express the partition function as a sum of imaginary-time, Euclidean path integrals, in which each path *x*(*t*) is assigned a statistical weight of exp[−*S*_E_(*x*)], with the Euclidean action *S*_E_(*x*), obtained from the action of the original problem, *S*_M_(*x*), often referred to as the Minkowski action, by inverting the potential *V*(*x*). Dynamics of a system driven by a bistable potential *V*(*x*) = *V*_b_(*x*) (blue line in Figure 2b) can thus be understood in terms of imaginary-time path integration with an action as dictated by a metastable potential *V*_m_(*x*) = −*V*_b_(*x*) (green line in Figure 2b).

When the initial and final states, |*x*_1_⟩and |*x*_2_⟩ (shown by red circles in Figure 2b), are separated by a potential barrier that exceeds the total energy of the system, the region under the barrier is forbidden for a classical particle (shaded area in Figure 2b). A search for the minimum of *S*_M_(*x*) then yields no stationary path *x*(*t*). Searching for the minimum of the Euclidean action *S*_E_(*x*), on the other hand, is still meaningful as it leads to Euler–Lagrange equations and respective equations of motion for a classical potential in the inverted potential (green line in Figure 2b). In such a potential, |*x*_1_⟩ is connected to |*x*_2_⟩ by a classical path (red dashed line in Figure 2b), whose *x*(*t*) map dominates path integration in eq 28, thus defining barrier-passage dynamics.

## 10 Recovering the Kramers Escape Rate

To reveal the connection of the above results to the Kramers escape rate, we consider a two-well bistable potential *U*_b_(*x*) of the general form (blue line in Figure 2c), not necessarily reducible to the special case of eq 20, along with its inverted, metastable counterpart *U*_m_(*x*) = −*U*_b_(*x*) (green line in Figure 2c). We focus on the eigenfunctions *ψ̅*_*n*_ (*x*) and eigenvalues *λ̅*_*n*_ that solve eq 10 for *U*(*x*) = *U*_m_(*x*) subject to absorbing boundary at *x* = ±*a*, where *a* is not necessarily finite, but is allowed to be infinite, as in the case of a generic *U*_m_(*x*) profile shown in Figure 2c. Such boundary conditions translate into reflecting boundary conditions for the bistable potential *U*_b_(*x*), obtained by inverting *U*_m_(*x*).^71^ One way to implement such boundary conditions in a physical, chemical, or biological system would be via particle removal, e.g., through an active transport chain or via a measurement, with a particle counter positioned at *x* = ±*a* to detect particles that cross the potential barrier.

For boundary conditions of this type, the eigenfunctions *ψ̅*_*n*_(*x*) and eigenvalues *λ̅*_*n*_ can be expressed through the eigenfunctions *ψ*_*n*_(*x*) and eigenvalues *λ*_*n*_ found by solving eq 10 with *U*(*x*) = *U*_b_(*x*) as^71,74^
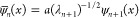
29and
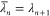
30As can be seen from eq 30, the lowest eigenvalue of the Fokker–Planck equation with a metastable potential *U*_m_(*x*), λ̅_0_, is equal to the lowest nonzero eigenvalue of the FP equation with a bistable potential, λ_1_. In a bistable potential, λ_1_, in its turn, defines the rate of transitions from one well of *U*_b_(*x*) to the other.

To relate these results to the Kramers escape rate, we modify the *U*_m_(*x*) potential curve in such a way as to provide an absorbing boundary at *x* = ±*a* with finite *a* (green dashed line in Figure 2c). With *a* chosen at a sufficiently large distance from the top of the barrier, such a modification of the potential can be considered a weak perturbation. An iterative calculation of the eigenvalue λ̅_0_ for *U*_m_(*x*) = −*U*_b_(*x*), with *U*_b_(*x*) as defined by eq 20, yields^71^ a perturbative series
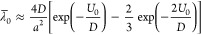
31This result recovers the exact expression eq 22 for the first nonvanishing eigenvalue of *Ĥ*_*FP*_ in a bistable potential as defined by eq 20 up to the order of exp(−3*U*_0_/*D*).

For a metastable potential *U*_m_(*x*) of a more general form (green line in Figure 2c), this iterative procedure gives
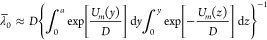
32For small *D*, the inner and outer integrals in eq 32 are dominated, respectively, by the minimum and the maximum of *U*_m_(*x*) (*x* = 0 and *x* = *x*_1_ in Figure 2b). With *U*_m_(*x*) expanded as power series about *x* = 0 and *x*_1_ in the inner and outer integrals, respectively, and with the limits of *y* extended to ±∞, integration in eq 32 yields
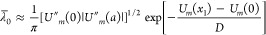
33

Comparing eq 33 to eq 4, and identifying *x* = 0 and *x*_1_ in eq 33 with respectively *x* = *x*_min_ and *x*_max_ in eq 4, we see that λ̅_0_ = *2r*_*K*_. A factor of 2 in this result reflects an obvious difference in barrier-passage settings. Unlike the original Kramers setting, which implies only one over-barrier escape pathway,^12,58^ the *U*_m_(*x*) profile of a metastable potential opens two escape pathways through two identical potential barriers (green line in Figure 2c), providing an escape rate twice as high as the Kramers escape rate *r*_K_.

## 11 Connecting to Hund’s Beat Cycle

To relate the eigenvalue λ_1_ as defined by eq 23 to the beat rate Γ_H_ = 1/*T*_H_ ∝ exp[−*V*_0_/(*ℏω*)] in Hund’s theory [eq 2], we expand the potential *V*(*x*) about the bottom of one of the potential wells of a bistable potential as *V*(*x*) ≈ *V*(*x*_*q*_) + *V*″*x*^2^/2, where *x*_*q*_ is the point where the potential reaches its minimum and *V″* is the second derivative of *V*(*x*) at *x* = *x*_*q*_. Choosing *V*(*x*_*q*_) as the level of zero energy, we express the potential barrier *V*_0_ as *V*_0_ ≈ *V″a*^2^/2, *a* being the half-width of the potential well. The oscillation frequency ω in eq 2 can now be expressed as ω^2^ ≈ 2*V*_0_/(*ma*^2^). The argument of the exponential in eq 2 thus becomes *V*_0_/(*ℏω*) ≈ (*mV*_0_/2)^1/2^*a*/*ℏ* ≈ *κd*, where *d* = *a*/2 and κ = (2*mV*_0_)^1/2^/*ℏ* are the decay length of the tunneling exponential under the barrier between the potential wells.

The potential *U*(*x*) in the argument of the exponential of λ_1_ in eq 23, on the other hand, can be written, with (*U′*)^2^/*D* ≫ *U″*, as
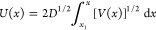
34where *x*_1_ is the classical turning point.

With *U*_0_ estimated from eq 34 as *U*_0_ ≈ 2*D*^1/2^*V*_0_^1/2^*L*, *D* = *D*_q_ = *ℏ*/2*m* and *L* identified as *d*/2, the argument of the exponential in eq 23 becomes *U*_0_/*D* ≈ *κd*, thus recovering the argument in the exponential of Hund’s beat cycle [eq 2], *V*_0_/(*ℏω*) ≈ (*mV*_0_/2)^1/2^*a*/*ℏ* ≈ *κd*.

## 12 The Keldysh Parameter: Euclidean-Action Insights

As one example, the Euclidean-action approach with its potential inversion offers important insight into the Keldysh γ_K_ parameter as defined by eq 5. Explaining the significance of γ_K_ as a parameter that controls laser-driven ionization, the opening paragraph of the seminal Keldysh paper^16^ argues in terms of “the tunneling time,” relating this parameter to the time τ_K_ = *d*_K_/*v* it takes for an electron to pass a barrier whose width is *d*_K_ = *I*_0_/(*eF*_0_) (Figure 1e). With the velocity of such an electron expressed as *v* = (2*I*_0_/*m*)^1/2^, the ionization rate, this argument points out, remains independent of the driver frequency as long as ω ≲ ω/γ = *eF*_0_/(2*mI*_0_)^1/2^, or, equivalently, in terms of the driver field cycle, *T*_0_ = 2π/ω, as long as *T*_0_ ≳ 4*πτ*_K_. Because the region under the potential barrier is classically forbidden, this interesting argument is open to interpretation. Specifically, the time τ_K_, often referred to as the “Keldysh tunneling time,” and its relation to the tunneling times that can be measured in ultrafast optical experiments,^97−100^ remains a subject of debates.^101,102^

As shown in the earlier work,^103−107^ τ_K_ can be related to the imaginary time, found by minimizing the action of an electron traversing the potential barrier. This approach turns out to be rather productive as it helps define important parameters of laser-induced ionization and allows the Keldysh photoionization theory to be extended to ultrashort laser pulses.^107−111^ Within the framework presented in this paper, the Keldysh tunneling time can be understood, very much in the spirit of the original Keldysh argument,^16^ by resorting to classical real-time dynamics driven by an inverted potential, −*V*_S_, in which the area under the barrier of *V*_S_ is classically allowed.

Indeed, in laser-induced ionization, the field of a laser driver, *E*_0_, modifies the potential that binds electrons in atoms and molecules, giving rise to a potential barrier *V*_K_(*x*) of a finite width, which can be estimated, following Keldysh,^16^ as *l* = *I*_0_/(*eF*). The region under this barrier is forbidden for a classical particle. Inversion of *V*_K_(*x*), however, transforms a potential barrier into a potential well (Figure 2b,c). In such a well, a ground-state electron will have a potential energy *I*_0_. Expanding the potential energy about the bottom of the well as *V*(*x*) ≈ −*V*_K_(*x*_0_) + *V″*_K_(*x*_0_)*x*^2^/2 and measuring the energy relative to −*V*_K_(*x*_0_), we find *I*_0_ ≈ *mω*_K_^2^*l*^2^/2, where ω_K_ = [*V″*_K_(*x*_0_)/*m*]^1/2^ is the oscillation frequency as dictated by the potential *V*(*x*) ≈ *V″*_K_(*x*_0_)*x*^2^/2. Plugging *l* = *I*_0_/(*eF*) into ω_K_ = (2*I*_0_/*m*)^1/2^*l*^–1^ gives ω_K_ = (2)^1/2^*eF*/(*I*_0_*m*)^1/2^. The ionization rate thus remains independent of the driver frequency ω as long as ω ≲ ω_K_ = (2)^1/2^*eF*/(*I*_0_*m*)^1/2^ = ω/γ, providing grounds for the Keldysh argument behind the γ parameter.

## 13 Low-Temperature Crossover from Thermally Activated Barrier Passage to Quantum Tunneling

At low temperatures, the thermal part of the diffusion coefficient, *D* = *D*_t_ = *kT*/(*mγ*), becomes small, suppressing the thermally activated barrier passage (purple arrow in Figure 1d). This tendency is readily seen from eq 1 for the rate of Arrhenius-reaction kinetics as well from eqs 4 and 33 for the Kramers escape rate. As thermally activated processes are suppressed, barrier passage is dominated by quantum tunneling^112−114^ across the potential barrier (red straight arrow in Figure 1d). As an early insight into a crossover from thermally activated to purely quantum barrier passage, Goldanskii et al.^115^ estimate the temperature of this crossover by equating the arguments in the Arrhenius-rate and tunneling exponentials [eqs 1 and 3], implicitly assuming that the activation energy *E*_a_ in the argument of the Arrhenius-law exponential in eq 1 is the same as the barrier height *V*_0_ in the tunneling exponential in eq 3. Although *E*_a_ in the Arrhenius-law exponential is generally not equal to *V*_0_ in the tunneling exponential, but, as we have shown above, relates to *V*_0_ via eqs 9 and 24, this approach is still useful as it provides a physically intuitive order-of-magnitude estimate for the crossover temperature: *T*_0_ ≈ (*ℏ*/*d*)[*V*_0_/(2*m*)]^1/2^, where *d* is the barrier half-width.

Important insights into a crossover from thermally activated barrier passage to quantum tunneling can be gained from the Euler–Lagrange equations, as dictated by the Euclidean action *S*_E_(*x*) for a metastable potential *U*_m_(*x*). At high *T*, *T* > *T*_0_, this equation allows only trivial solutions for *x*(*t*), each describing a system located in one of the potential wells of the original bistable potential *U*_b_(*x*) = −*U*_m_(*x*) (Figure 2b). For *T* below *T*_0_, however, a nontrivial periodic solution *x*(*t*) = *x*_e_(*t*), often referred to a bounce solution,^117−120^ is also allowed. Because the action *S*_E_(*x*) for this solution is smaller than that of *S*_E_(*x*) for the trivial solutions, *x*_e_(*t*) tends to dominate low-temperature barrier-passage dynamics.

An instructive picture of interplay between thermally activated barrier passage and quantum tunneling can be drawn from Hopfield’s distribution function,^35^ derived for electron transfer processes in biological systems. In Hopfield’s distribution, the temperature-dependent factor, β = 1/(*kT*), in the argument of exponentials describing the rates of thermally activated barrier-passage processes, such as the rate of Arrhenius-law kinetics [eq 1], Kramers escape rate [eq 4], as well as the rate at which a diffusion-driven statistics universally converges to the Gibbs distribution [eqs 15 and 24], is replaced by
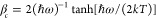
35where *ℏω* is the energy of the relevant vibrational coordinate.

At high temperatures, *T* ≫ *ℏω*, tanh[*ℏω*/(2*kT*)] ≈ *ℏω*/(2*kT*), leading to β_c_ → β = 1/(*kT*), thus recovering the temperature-dependent exponential of purely classical thermally activated barrier passage. In the low-temperature limit, *T* ≪ *ℏω*, on the other hand, tanh[*ℏω*/(2*kT*)] ≈ 1 and β_c_ → 2/(*ℏω*).

We can now see that, at low temperatures, *T* ≪ *ℏω*, the exponential exp(−*U*_0_/*D*), which, in accordance with eqs 4, 24, and 33, governs the rates of thermally activated barrier-passage processes, becomes the Hund’s beat rate exponential, exp[−*V*_0_/(*ℏω*)]. In a suitable physical setting, this exponential, as we have shown above [see, e.g., eqs 34], is equivalent to the tunneling exponential of eq 3. Hopfield’s model of electron transfer in biological systems thus suggests a continuous crossover from the high-temperature regime of barrier-passage dynamics, dominated by thermally activated processes, to the low-*T* regime, where quantum tunneling plays a dominant role.

## 14 Conclusion

To summarize, the model of a stochastic Markovian process has been shown to provide a unified framework for the description of a vast class of classical and quantum barrier-passage phenomena, revealing an innate connection between various types of barrier-passage dynamics and providing closed-form equations showing how the signature exponentials in classical and quantum barrier-passage rates relate and translate into each other. In this framework, the Arrhenius-law kinetics, the emergence of the Gibbs distribution, Hund’s molecular wave packet well-to-well oscillatory dynamics, Keldysh photoionization, and Kramers’ escape over a potential barrier are all understood as manifestations of a potential-driven Markovian dynamics whereby a system evolves from a state of local stability. At low temperatures, the temperature-dependent factor β = 1/(*kT*) in the argument of exponentials describing the rates of thermally activated barrier-passage processes is shown to continuously transform into a tunneling exponential. The Keldysh adiabaticity parameter and the Keldysh tunneling time are understood in this framework in terms of classical real-time dynamics driven by an inverted potential, – *V*_S_, in which the area under the barrier of *V*_S_ is classically allowed.
